# Proteomic Profiling of Aqueous Humor Exosomes from Age-related Macular Degeneration Patients

**DOI:** 10.7150/ijms.73489

**Published:** 2022-05-13

**Authors:** Ching-Yao Tsai, Chueh-Tan Chen, Hsin-Han Wu, Chen-Chung Liao, Kate Hua, Chung-Hua Hsu, Chian-Feng Chen

**Affiliations:** 1 Department of Ophthalmology, Taipei City Hospital Zhongxing Branch, Taipei, Taiwan; 2 Institute of Public Health, National Yang Ming Chiao Tung University, Taipei, Taiwan; 3 Department of Business Administration, Fu Jen Catholic University, New Taipei City, Taiwan; 4 Institute of Traditional Medicine, National Yang Ming Chiao Tung University, Taipei, Taiwan; 5 Cancer Progression Research Center, National Yang Ming Chiao Tung University, Taipei, Taiwan; 6 Metabolomics-Proteomics Research Center, National Yang Ming Chiao Tung University, Taipei, Taiwan; 7 Department of Chinese Medicine, Taipei City Hospital, Linsen, Chinese Medicine, and Kunming Branch, Taipei, Taiwan

**Keywords:** Aqueous Humor, Age-related macular degeneration, Exosome

## Abstract

**Purpose:** The alteration of the exosomal proteins in the aqueous humor (AH) is linked to the development of eye diseases. The goal of this study was to examine the exosomal protein profile of patients with age-related macular degeneration (AMD) to better understand their role in the pathogenesis of AMD.

**Methods:** Exosomes were isolated from the AH of 28 AMD and 25 control eyes. The quality, concentration, and size distribution of exosomes were measured using a nanoparticle tracking analysis system (NTA). Total exosomal proteins from each sample were purified and digested with trypsin for liquid chromatography-tandem mass spectrometry (LC-MS/MS) analysis.

**Results:** Based on LC-MS/MS analysis, we got 105 exosomal peptides from AMD and control patients. Gene ontology (GO) analysis in the biology process revealed that exosomal proteins of AMD were enriched in the lipoprotein metabolic process. T-test analysis revealed six exosomal proteins in patients with AMD were significantly different from controls. Comparing the exosomal protein profile of AMD patients who were receiving anti-VEGF therapy, we observed the amount of two proteins decreased with the duration of the anti-VEGF treatment time.

**Conclusions:** In this study, we successfully isolated and purified AH exosomes. Our results provide pioneering findings for the exosomal protein profile in AMD development and under therapy. These unique proteins could be the new targets for drug discovery or biological markers for evaluating therapeutic efficacy.

## Introduction

The macula is a pigmented area of the retina that is responsible for sharp, clear, straight-ahead vision. As people get older, structure and blood flow changes in the macula cause age-related macular degeneration (AMD). AMD is one of the leading causes of irreversible vision loss worldwide 1. The most significant risk factor of AMD is aging. Almost all patients with advanced AMD are over the age of 60 2. Genetic factors are also important, and polymorphisms in over 30 genes have been reported to be associated with increased risk of AMD 3. Others risk factors such as smoking, uncontrolled hypertension, body mass index above 25 have been reported in previous studies 4-7.

AMD begins with the formation of drusen, which are lipoprotein-rich deposits in the macula. The size of the drusen is linked to the severity of AMD 8. Patients with early AMD are usually asymptomatic or have decreased vision, distortion or blind spots in or around their central vision. Patients in advanced stages will have difficulty recognizing faces, driving, reading, or performing other daily activities dues to loss of central vision 9. In general, advanced AMD is classified as wet and dry base on the presence or absence of abnormal blood vessels growing beneath the retina, resulting in blood or serum leakage. The disease process of dry AMD is slower than wet AMD, and visual acuity is better preserved 10. For the pathogenesis of AMD is still largely unknown, the current treatments for AMD are aimed at delaying or controlling the disease progression. For example, wet AMD can be treated with intraocular injections of anti-VEGF drugs to slow down neovascularization and retinal thickening 11. For the limited options for prevention and treatment of AMD, to explore new therapeutic targets and biomarkers for AMD is important.

Exosomes are made up of a lipid bilayer that contains transmembrane proteins, cytosolic proteins, and RNAs 12. They are secreted from cells and are released into body fluids (such as blood, urine, tears, and spinal fluid), which can then be transported between cells via the circulatory system. As a result, they are regarded as critical messengers for cell-to-cell communication 13. Aqueous humor (AH) is the clear liquid filling in the anterior and posterior chamber of the eye 14. Lines of evidence suggest abundant exosomes are common in AH 15, 16. Recent research has linked exosomal proteins or miRNAs to variety of ophthalmic diseases 17-20. To examine the changes of exosomal proteins that occur with the development of AMD, we aimed to compare the individual protein profiles from patients with AMD, using liquid chromatography-tandem mass spectrometry (LC-MS/MS).

## Materials and Methods

### AH Samples Collection

The Research Ethics Committee of Taipei City Hospital (TCHIRB-10712118) approved the study protocol, which was carried out by the tenets of the Helsinki Declaration. Before enrolling in the study, all participants provided written informed consent. Human AH samples were collected from patients undergoing cataract surgery at the Zhongxing branch of Taipei City Hospital. The patients with AMD were confirmed by the use of fluorescein angiography. The control eyes came from senile cataract patients who did not have other ocular or systemic diseases. Patients who received three consecutive intravitreal injections of ranibizumab (Lucentis, 10 mg/ml, Novartis, Basel, Switzerland) were asked to provide their AH during anti-VEGF treatment. Approximately 100 μL of AH was collected from each patient via anterior chamber paracentesis with a needle inserted through the peripheral cornea at the start of the procedure. Undiluted AH samples were collected in sterile tubes and stored at - 80°C until further investigation.

### Isolation of Exosomes from the AH

Exosomes were isolated from the AH using the ExoQuick precipitation solution (EXOQ5A-1, System Biosciences, Inc., Mountain View, CA) by the manufacturer's instruction. Approximately 100 μl of AH was centrifuged at 3000  ×  g for 15 minutes to remove cellular debris and collected the supernatants. PBS buffer (10010023, Thermo Fisher Scientific; Waltham, MA) was added to the supernatants to make a final volume of 250 μl. After that, 63 μl of precipitation solution was added to the supernatants and incubated overnight. The exosome pellets were separated by centrifugation at 12,000  ×  g for 90 minutes and suspended in 100 μl PBS. The concentration and size distribution of the vesicles was assessed by NanoSight LM10 (LM10, Malvern Instruments, Rancho Cucamonga, CA).

### Proteins Purification and LC-MS/MS Analysis

To extract the exosomal proteins, exosomes were lysed by RIPA buffer (89900, Thermo Fisher Scientific; Waltham, MA). The proteins were then digested with trypsin (60109-101, SMART Digest Trypsin Kit, Thermo Fisher Scientific; Waltham, MA), desalted (Z720070, Millipore Ziptips Micro-C18; Sigma-Aldrich, Milwaukee, WI), purified (60309-001, SOLA™ SPE Plates; Thermo Fisher Scientific; Waltham, MA), and dissolved in 0.1% formic acid for LC-MS/MS analysis (LTQ Orbitrap Velos, Thermo Scientifics; Waltham, MA) (service provided by the Mass Core Facility of Genomics Research Center, Academia Sinica). The acquired proteomics raw data files were then searched against a UniProt human protein database (http://www.uniprot.org/) by using PEAKS Studio 7.5 (PEAKS Studio, Bioinformatics Solutions, Waterloo, Ontario, Canada). The following settings were used in PEAKS Studio 7.5 in conjunction with UniProt to search the protein database: enzyme set to trypsin with a maximum of two missed cleavage site precursor and fragment mass tolerance of 20 ppm and 0.8 Da, respectively. Finally, the spectral counts obtained from each peptide were normalized to the total spectral counts recorded for all peptides in a sample.

### Statistical Analyses

Data are expressed as the mean ± standard error of the mean. A paired sample t-test was used to analyze the axial length and exosome concentration measurements. SPSS version 24 was used to analyze all of the data (SPSS, Inc; Chicago, IL). A P value of less than 0.05 was considered to display a statistically significant difference. The principal component analysis (PCA) plot of all samples was generated using Partek Genomics Suite 7.18 (Partek Genomics Suite, Partek Inc., St. Louis, MO). Gene ontology enrichment analysis was conducted by ShinyGO v0.75 (http://bioinformatics.sdstate.edu/go/)^21^.

## Results

### Samples Collection and Exosomes Isolation

**Figure 1:**
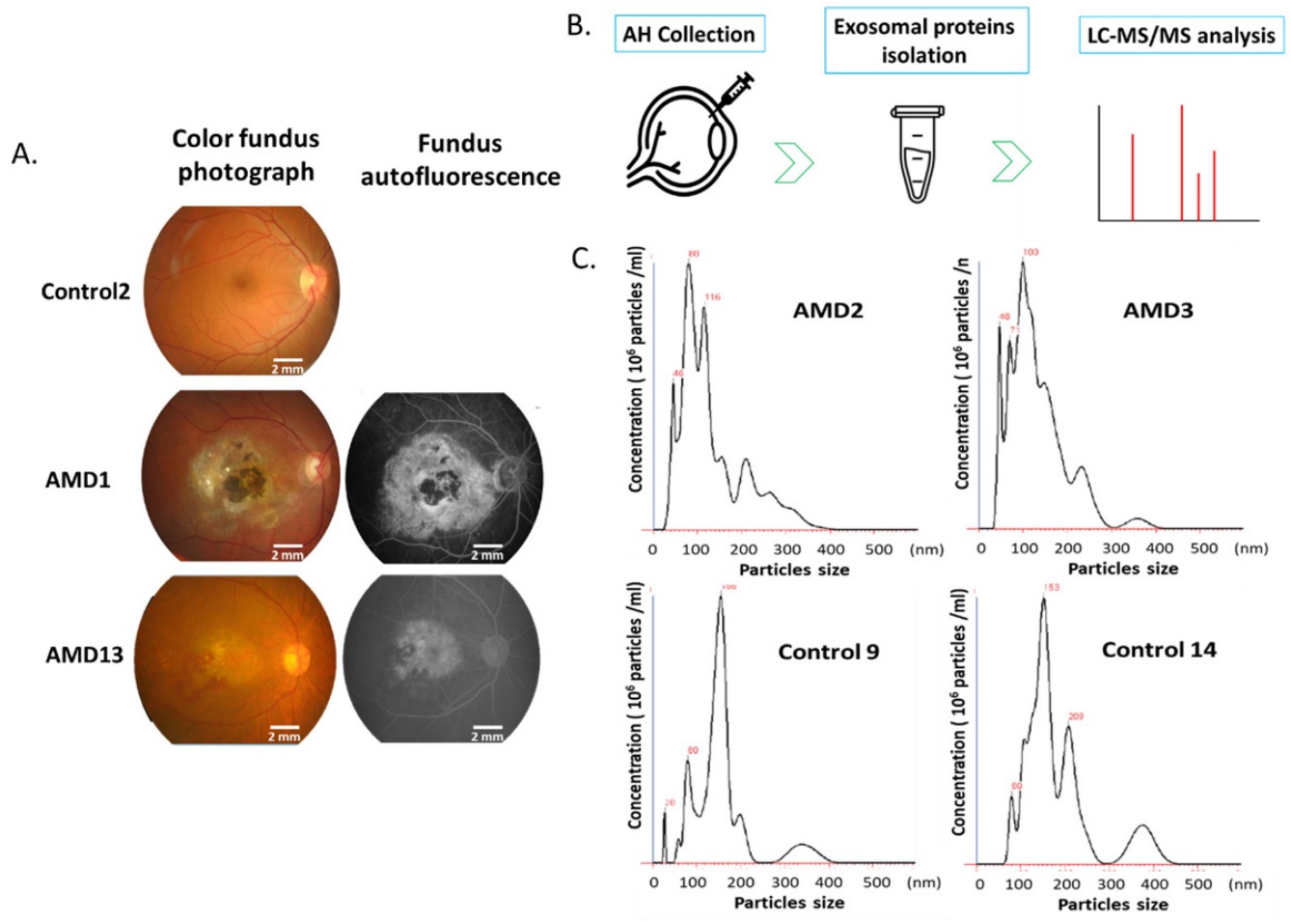
** Ocular examination and exosomes isolation.** A) Color and Fundus autofluorescence imaging for AMD eyes. B) Representative nanoparticle tracking analysis of exosomes isolated from the AH of patients with AMD and controls.

In this study, all the patients with AMD were confirmed by fluorescein angiography (Fig.1A). AH samples were collected from 28 AMD and 25 control eyes. Exosomes were isolated from each sample using the exosome purification kit. The concentration and size distribution of purified small vesicles were determined by nanoparticle tracking analysis (NTA) system. At first, the camera of NTA captures a video of all particles moving under Brownian motion. The NTA software tracks the random thermal motion of each particle to determine the diffusion coefficient which is used to calculate the size of each particle. Since NTA allows the determination of size distribution from 10 to 1000 nanometers (nm) of particles, it is one of the prominent technologies used for high-throughput analysis of individual exosomes 22. By the analysis of NTA, most of purified exosomes were in the expected size range (Fig. 1B).

### Exosomal Proteins Isolation and LC-MS/MS Analysis

**Figure 2:**
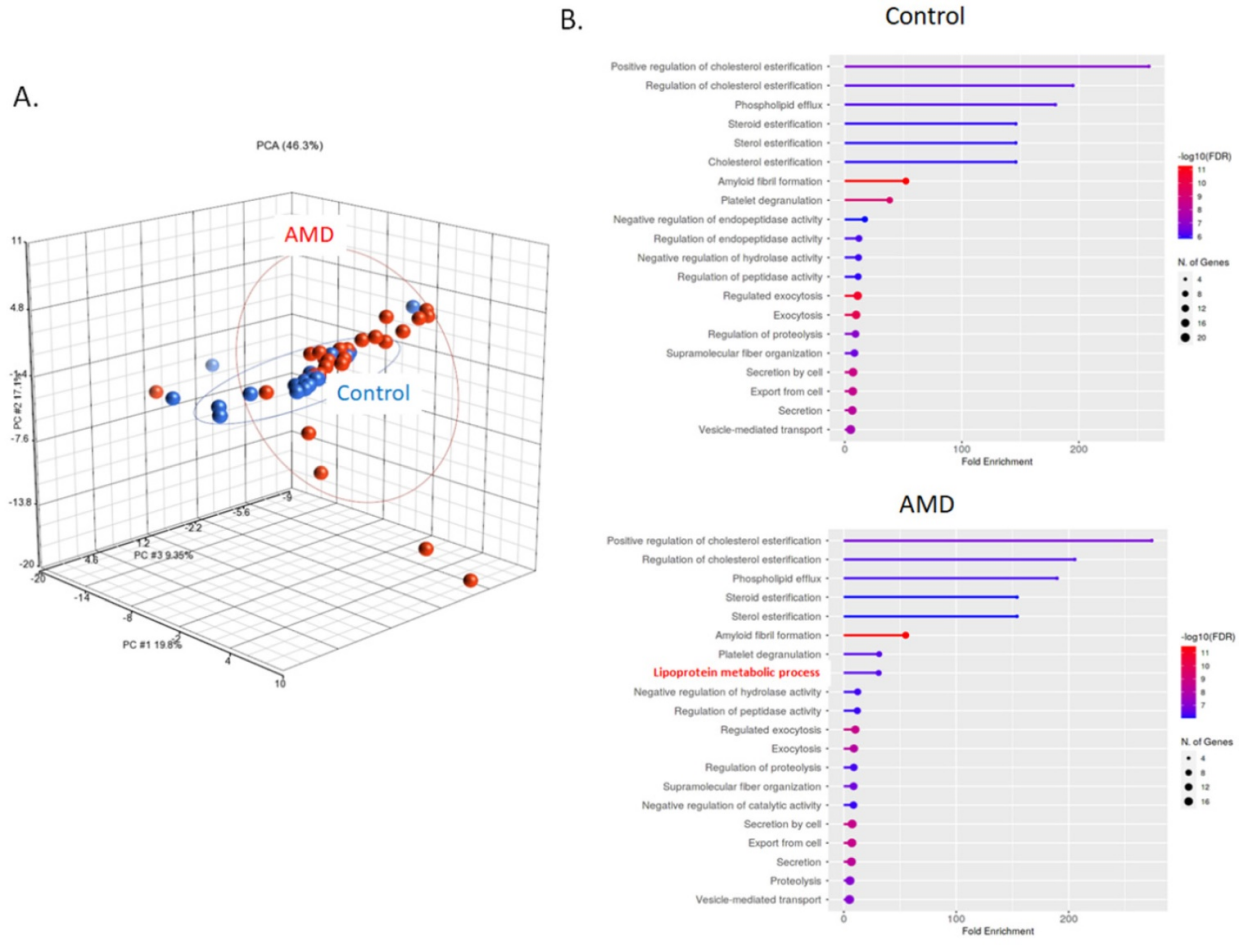
** LC-MS/MS identified peptides from AMD and control.** A) Common and unique identified peptides from individual AMD and control patients. B) The PCA plot of all samples was generated to assess the variability of peptide expression in myopia and control patients. (red, AMD; blue, control). C) Gene ontology enrichment analysis in biology process for AMD and control group.

Exosomal proteins were extracted and digested with trypsin for LC-MS/MS quantitation analysis. The information about the peptides was obtained by aligning the peptide sequences to the UniProt database (www.uniprot.org). Principal component analysis (PCA) was applied to investigate internal variation between AMD and control groups (Fig. 2A). According to the PCA results, the AMD and control groups partially overlapping, but high variation was observed between individual of AMD. The analysis of gene ontology (GO) enrichment showed that most of the gene-set in the biology process were similar in the control and AMD. A gene-set, lipoprotein metabolic process, was only enriched in AMD (Fig. 2B). Since lipid metabolism is one of the important pathways in AMD pathogenesis 23. It would be interesting to further explore their roles in cell-to-cell communication during the pathogenesis of AMD.

### AMD-Specific Exosomal Proteins

**Figure 3:**
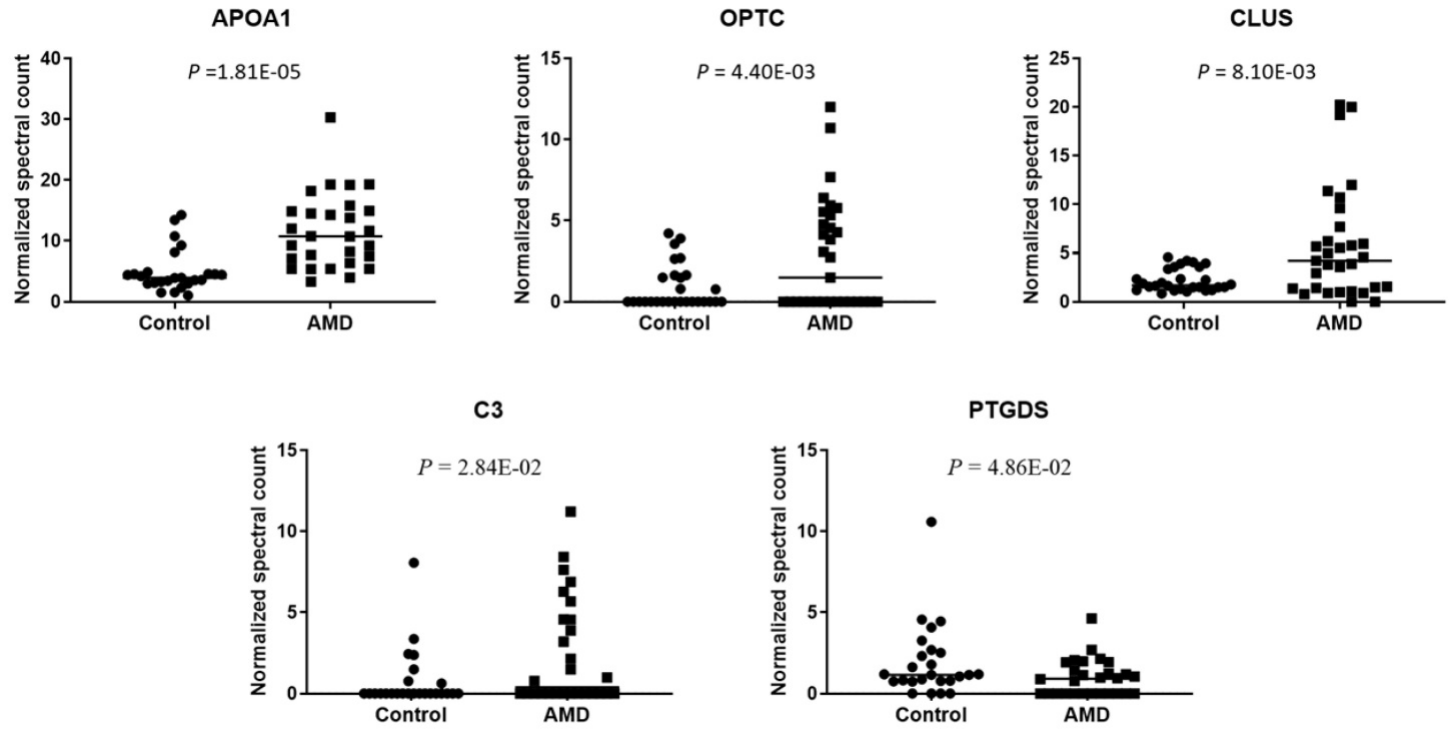
** Normalized spectral count (NSC) of AMD-specific proteins were detected from LC-MS/MS.** Scatterplot presenting the NSC of proteins detected in each patient from AMD or control. *P* values were calculated by paired two -tail *t-*tests.

To compare the level of exosomal proteins between in AMD and control, the spectral counts obtained from each peptide were normalized to the overall spectral counts recorded for all peptides in a sample. Ten peptides were found to be significantly different between the AMD and the control (t-test, *P* < 0.05) (Table 1). These peptides are from 5 known proteins and 1 unidentified protein (Fig. 3). In previous studies, some of them were found to be associated with AMD. APOA1 is the main component of HDL particles 24. The presence of APOA1 in plasma and urine has been linked to an increased risk of AMD 25-27. CLU has been found in the drusen of patients with AMD 28. According to the report from Kim. et. al, CLU may protect oxidative stress-induced apoptosis in retinal pigment epithelial cells 29. C3 is one of the most important components of the complement system. Elevated plasma levels of C3 are associated with an increased risk of AMD 30, 31. To the best of our knowledge, we are the first to identify these proteins in the exosome of AH.

### Exosomal Protein profile in anti-VEGF treatment AMD

**Figure 4:**
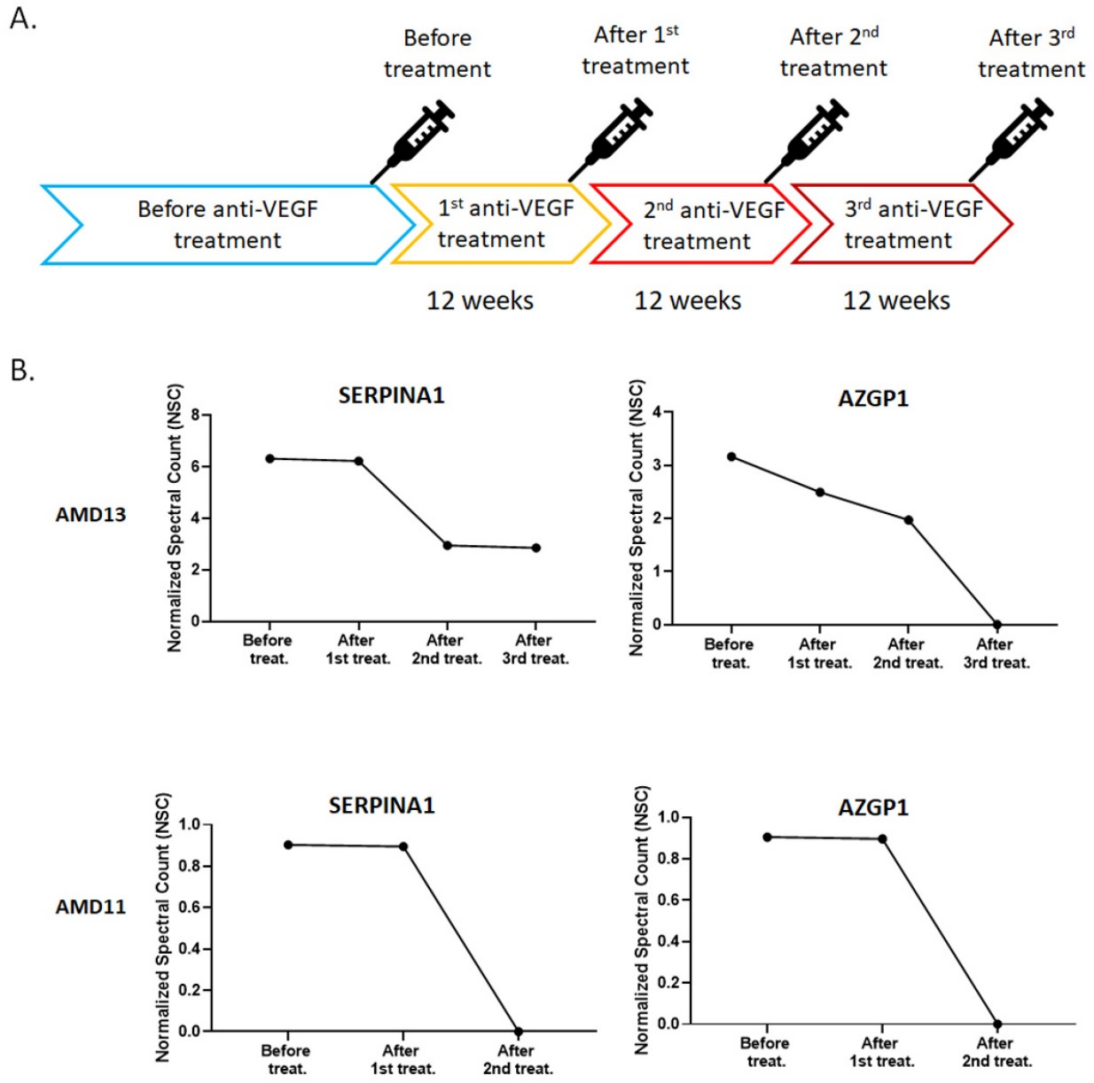
**Normalized spectral count (NSC) of exosomal proteins that were decreased during continuous anti-VEGF injections.** A) The Flow chart of the anti-VEGF therapy process. B) The NSC of proteins detected before and after anti-VEGF therapy in different time points.

Under normal physiological conditions, the expression of VEGF is relatively low in retinal tissues. On the other hand, the expression of VEGF will increase significantly in a condition of ischemia, hypoxia, or inflammation 32. Evaluation of VEGF level cause blood vessels growth and leakage, which is the primary cause of wet AMD 33. Based on this theory, anti-VEGF therapies are commonly used to treat with the wet AMD 34. The patients must be injected with the anti-VEGF drugs into the eye for every four to twelve weeks depending on their response 34. For the exosome content is common response to external stimuli, we were interested in the change in exosomal protein profile under continuous anti-VEGF drug treatment. The AH was collected from two patients who had received continuous anti-VEGF injections of ranibizumab every 12 weeks (Fig. 4A). Comparing their exosomal proteins before and after treatment, we found that the amounts of two proteins, SERPINA1 and AZGP1, were decreased with anti-VEGF treatment time (Fig. 4B).

## Discussion

In this study, we used LC-MS/MS to examine the exosomal proteins from the AH of AMD by LC-MS/MS. Comparing the protein profile of AMD and control, six proteins showed significant enrichment in patients with AMD. Three of them, APOA1, CLU, and C3, have been suggested to be linked with AMD in previous studies. To the best of our knowledge, we are the first to show that these proteins were enriched in exosome of AMD eyes. Anti-VEGF treatments are used to control the progress of wet AMD. By comparing before and after treatment, we found two exosomal proteins, SERPINA1 and AZGP1, were decreased with treatment time. As a result, we hypothesized that VEGF induced these proteins to promote AMD development. More research is needed to investigate their roles in the pathology of AMD.

Elevated HDL cholesterol levels are important in the development of AMD since drusen is composed by lipids 35. APOA1 is one of the major components of HDL. The link between plasma level of APOA1 and the risk of AMD has been established in previous studies 25-27. The complement system is frequently activated in many inflammatory diseases including AMD 36. As key components of the complement cascade, genetic variations 37 and elevated plasma levels of C3 30, 31 are associated with the risk of AMD. Our findings suggested that exosomal APOA1 and C3 are higher in AMD eyes. This raises the intriguing possibility that APOA1 and C3 in plasma could also be packaged into exosome and then cross the blood- retina barrier (BRB). BRB made up of cells that are tightly packed together to prevent uncontrolled leakage of substances such as ion, protein, and water into and out of the retinal 38. Recently, increasing evidence suggested exosome can cross biological barriers such as the blood- brain barrier (BBB) 39. *In vivo* experiments are necessary to validate our hypothesis. The pathogenesis of AMD is thought to be influenced by oxidative stress. Increased oxidative stress stimulates CLU as a physiological defense to maintain cell viability 40. *In vitro* studies suggested that CLU could inhibit oxidative stress-induced caspase-3 activity, thereby protecting retinal pigment epithelial cells 29. As a result, CLU could be considered as a preventive approach for AMD. The role of AMD-specific exosomal proteins in the pathogenesis of AMD still need to be proved. We plan to observe the changes in cell physiology and gene expression which caused by the uptake of these exosomal proteins.

Anti-VEGF therapy is used to control the progression of wet AMD. However, some patients have a poor response or lose the efficacy to anti-VEGF agents after repeated administration 41. Comparing the exosomal protein profile before and after anti-VEGF treatment, the level of SERPINA1 and AZGP1 in the exosome was significantly decreased. VEGF is known to stimulate endothelial cell proliferation, migration, new vessel formation, and ultimately leading to angiogenesis 42. Previous research has shown that SERPINA1 promotes cell migration and invasion 43, 44. AZGP1 could improve cell proliferation and epithelial-mesenchymal transition (EMT) 45, 46. For the SERPINA1 and AZGP1 were decreased with anti-VEGF treatment time, we proposed they may under the regulation of VEGF and could be biomarkers for curative effect of anti-VEGF therapy in AMD. Due to the time-consuming for sample collection, only two patients were shown in this study. Expanding the number of samples to verify the association of SERPINA1 and AZGP1with anti-VEGF therapy is our next goal.

In conclusion, this study revealed the unique exosomal proteins in AMD patients and receiving anti-VEGF therapy patients. These proteins shed light on potential new targets for the diagnosis and treatment of AMD.

## Figures and Tables

**Table 1:** Significantly changed exosomal proteins in AMD

Protein IDs	Protein Name	Gene Name	Control (n = 25)	AMD (n = 28)	*p*-value
			Mean ± SEM	Mean ± SEM	
P02647	apolipoprotein A1	APOA1	4.991 ± 0.683	11.574 ± 1.158	1.81E-05
A0A024R3E3	apolipoprotein A1	APOA1	4.991 ± 0.683	11.574 ± 1.158	1.81E-05
Q6MZU6	Putative uncharacterized protein DKFZp686C15213		0.903 ± 0.211	3.103 ± 0.636	2.94E-03
Q9UBM4	opticin	OPTC	0.526 ± 0.171	2.069 ± 0.463	4.40E-03
P10909	clusterin	CLU	2.032 ± 0.205	5.291 ± 1.101	8.10E-03
V9HWA9	complement C3	C3	0.766 ± 0.350	2.420 ± 0.615	2.84E-02
P01024	complement C3	C3	0.766 ± 0.350	2.420 ± 0.615	2.84E-02
P41222	prostaglandin D2 synthase	PTGDS	1.938 ± 0.442	0.968 ± 0.211	4.86E-02
A0A024R8G3	prostaglandin D2 synthase	PTGDS	1.938 ± 0.442	0.968 ± 0.211	4.86E-02

## Abbreviations

AH: aqueous humor
AMD: Age-related macular degeneration
LC-MS/MS: liquid chromatography-tandem mass spectrometry
PCA: principal component analysis
